# Social factors associated with non-suicidal self-injury (NSSI)

**DOI:** 10.1186/s13034-019-0284-1

**Published:** 2019-06-12

**Authors:** Rebecca C. Brown, Andreas Witt

**Affiliations:** Department of Child and Adolescent Psychiatry and Psychotherapy, University Hospital Ulm, Steinhoevelstr. 5, 89075 Ulm, Germany

Non-suicidal self-injury (NSSI) is highly prevalent among adolescents and young adults. Besides (neuro-) biological elements, social factors seem to play a crucial role in the onset and maintenance of NSSI. These factors can include parent–child or sibling relationships, peer-relationships, as well as experiences with social media or at school. Although cultural differences may have a large influence on the prevalence and nature of NSSI, little is known about NSSI in non-Western countries. Furthermore, potentially traumatizing life events can be associated with the onset and maintenance of NSSI. Especially for young people who were forced to leave their homes and live as refugees in foreign countries, one might expect higher rates of NSSI. However, research on NSSI in this particularly vulnerable group is scarce.

In the current thematic series we brought together six unique manuscripts from different cultural backgrounds, focusing on a variety of potential social risk factors for the onset and maintenance of NSSI.

The first manuscript by Lauw et al. [1] describes the prevalence, nature and risk factors for deliberate self-harm among adolescents in Singapore. Interestingly, the prevalence rate of 23.1% was similar to results of studies from Western countries, and the most common type of NSSI (cutting) corresponds to Western reports. Furthermore, NSSI was associated with depressive symptoms, female gender, and alcohol use, as has been shown in previous studies. However, unlike previous studies from Western cultures, NSSI was not associated with family factors. The authors suggest that this might be due to stigma and common misperceptions towards mental illness in Singapore, which may have led to participants withholding information about a positive history of psychiatric illness within the family. Future research from Asian countries is needed in order to find out more about social and other risk factors in Asian cultures.

Another manuscript focusing on adolescents from non-Western countries by Verroken et al. [2], reports on NSSI among refugee minors in Belgium. Unexpectedly, prevalence rates, methods and functions of NSSI are comparable to results from Western study samples. However, those adolescents who engaged in NSSI showed a rather high number of acts of NSSI as compared to Western studies, as well as an increased risk of clinically significant emotional, conduct, and peer problems, and suicidality. Interestingly, like in the Singaporean study [1], family factors (i.e. being accompanied or not, having both parents around), were not significantly associated with NSSI.

Results from those two studies are especially remarkable in the light of the other four manuscripts of this series. These studies were conducted in Western samples and focused on family factors associated with NSSI.

Two longitudinal studies investigated the association with peer and parent relationships and NSSI. The first study by Victor et al. [3] was conducted in an urban sample of US-American adolescent girls. In this study, negative parental variables (like parental harsh punishment, low parental monitoring and poor quality of attachment to parents) predicted NSSI onset, while positive parenting behaviors decreased the odds of NSSI. Similarly, negative peer variables (like poorer social self-worth and self-competence, more negative perception of peers and peer victimization) increased the odds for NSSI. Furthermore, in combined multivariate models, only peer factors were associated significantly with NSSI onset. This study therefore shows the importance of both peer and parental relationships in the light of NSSI onset.

The second study on this matter by Gandhi et al. [4] investigated longitudinal data from a high school data set from Belgium. In addition to showing a significant association of peer and maternal attachment with subsequent NSSI, this study showed a significant mediation of this association by identity synthesis and confusion. As also discussed by Victor and colleagues, it therefore seems to be important to look at factors that are influenced by dysfunctional paternal or peer relationships or attachment, like identity formation, which then in turn might lead to onset or ongoing NSSI.

While parents and peers are often targeted when investigating mental illness, siblings are mainly overlooked. In the first study of its kind, Tschan et al. [5] investigated sibling relationships of Swiss female adolescents in the light of NSSI. Adolescents with NSSI reported significantly higher rivalry scores and less warmth and empathy in sibling relationships than non-clinical controls. Furthermore, when siblings of adolescents with NSSI were asked, they also reported a wide range of negative emotional and familial consequences of their sisters’ NSSI. For example, they reported to be feeling left alone or a distressing family situation. This study highlights the importance of considering the whole family system, not only with regard of risk or protective factors for the onset or maintenance of NSSI, but also with regard of possible negative impacts on other family members.

The last (but certainly not least) study of this series by Waals et al. [6] describes the NSSI Family Distress Cascade Theory, which addresses the most important points of the previous manuscripts included in this series. It explains how adolescents go through a challenging period between personal autonomy and connectedness with their family, and identity formation. When NSSI occurs, caregivers often feel confused about how to react to their child’s NSSI, as they may feel ashamed, afraid or shameful. In reaction to those feelings, caregivers might increase efforts to control the child’s behavior. In turn, the adolescent might experience this behavior as an intrusion, which might then lead to decreased family functioning, which might then lead to increased risk for NSSI (as shown by other articles in this series).

In summary, manuscripts of this series show how complex the association of social factors and NSSI is. First, there seem to be cultural differences with regard of the perceived impact of family factors on NSSI. While all studies conducted in Western samples showed associations of family factors with NSSI, those conducted in non-Western studies did not. Whether this is due to actual differences, or differences in how cultural norms influenced participants’ answers, is yet to be determined. Next to the impact of negative peer and paternal relationship on NSSI onset in two longitudinal studies, two other manuscripts made a strong point of considering the whole family system and keeping the needs of psycho-education and sometimes also psychotherapy of parents and siblings of adolescents with NSSI in mind.

## Data Availability

None.
